# Sacituzumab govitecan, an antibody‐drug conjugate targeting trophoblast cell‐surface antigen 2, shows cytotoxic activity against poorly differentiated endometrial adenocarcinomas *in vitro* and *in vivo*

**DOI:** 10.1002/1878-0261.12627

**Published:** 2020-01-14

**Authors:** Emanuele Perrone, Paola Manara, Salvatore Lopez, Stefania Bellone, Elena Bonazzoli, Aranzazu Manzano, Luca Zammataro, Anna Bianchi, Burak Zeybek, Natalia Buza, Joan Tymon‐Rosario, Gary Altwerger, Chanhee Han, Gulden Menderes, Gloria S. Huang, Elena Ratner, Dan‐Arin Silasi, Masoud Azodi, Pei Hui, Peter E. Schwartz, Giovanni Scambia, Alessandro D. Santin

**Affiliations:** ^1^ Department of Obstetrics, Gynecology, and Reproductive Sciences Yale University School of Medicine New Haven CT USA; ^2^ Department Woman and Child Health Sciences Universita' Cattolica del Sacro Cuore Rome Italy; ^3^ Department of Experimental and Clinical Medicine Magna Graecia University Catanzaro Italy; ^4^ Department of Pathology Yale University School of Medicine New Haven CT USA

**Keywords:** antibody–drug conjugate, endometrial carcinoma, IMMU‐132, sacituzumab govitecan, uterine cancer

## Abstract

Endometrial cancer is the most common gynecologic malignancy in developed countries. The antibody–drug conjugate (ADC) sacituzumab govitecan (SG) targets trophoblast cell‐surface antigen‐2 (Trop‐2) – a cell‐surface glycoprotein highly expressed in many epithelial tumors – and delivers the active metabolite of irinotecan SN‐38 to Trop‐2‐positive tumor cells. We evaluated Trop‐2 expression in endometrial endometrioid carcinoma (EC) tissues and the activity of SG against primary poorly differentiated EC cell lines and xenografts. Trop‐2 expression was assessed in 143 formalin‐fixed–paraffin‐embedded tumors and seven primary tumor cell lines by immunohistochemistry and flow cytometry, respectively. Cell viability of primary tumor cell lines was assessed following exposure to SG, or control antibodies. Antibody‐dependent cell cytotoxicity (ADCC) against Trop‐2‐positive and Trop‐2‐negative EC cell lines was measured *in vitro* using 4‐h chromium release assays. A Trop‐2‐positive EC xenograft model was used to determine the *in vivo* activity of SG. Moderate‐to‐strong staining was detected in 84% (120/143) of EC samples, whereas 43% (3/7) of the primary EC cell lines tested overexpressed Trop‐2. EC cell lines overexpressing Trop‐2 were significantly more sensitive to SG compared to control ADC (*P* = 0.014 and *P* = 0.005). Both SG and the unconjugated parental antibody hRS7 mediated high ADCC against Trop‐2‐positive cell lines. Moreover, SG induced significant bystander killing of Trop‐2‐negative tumors cocultured with Trop‐2‐positive tumors. In the xenograft model, intravenous administration of SG twice weekly for three weeks was well tolerated and demonstrated impressive tumor growth inhibition against poorly differentiated, chemotherapy‐resistant EC xenografts (*P* = 0.011). In summary, SG is a novel ADC with remarkable preclinical activity against poorly differentiated EC cell lines overexpressing Trop‐2. These findings warrant future clinical trials.

## Graphical abstract

TROP‐1 is overexpressed in the majority of endometrial cancers. Sacituzumab Gavitecan (SG) is highly effective, both in vitro as well as in vivo, against primary endometrial cancer cell lines and xenografts overexpressing TROP‐2. Clinical studies with SG in patients with recurrent, chemotherapy‐resistant endometrial cancer overexpressing TROP‐2 are warranted.

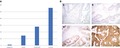

**Abbreviations**
ADC: antibody–drug conjugate
ADCC: antibody‐dependent cellular cytotoxicity
control ADC: nontargeting control antibody–drug conjugate
DAR: drug‐to‐antibody ratio
EC: endometrial endometrioid carcinoma
hRS7: unconjugated monoclonal antibody
PBLs: peripheral blood lymphocytes
SG: sacituzumab govitecan
SCID: severe combined immunodeficiency
TMA: tissue microarray
Trop‐2: trophoblast cell‐surface antigen‐2

## 1 Introduction

Endometrial endometrioid carcinoma (EC) is the most prevalent gynecologic malignancy in the United States, with approximately 61 880 newly diagnosed cases and 12 160 deaths in 2019. Unlike ovarian and cervical cancer, the incidence and mortality rates from EC are globally increasing. If current trends continue, the incidence of EC in the United States will double by 2030 (Siegel *et al.*, 2019).

Traditionally, histologic classification has been used to counsel patients regarding prognosis and to guide decision‐making regarding the necessity for adjuvant treatment. Women diagnosed with early‐stage well‐differentiated endometrioid endometrial cancers have a 5‐year overall survival rate of over 90% after surgery with or without adjuvant radiation (Felix *et al.*, 2010). However, up to 35% of EC patients are diagnosed with a more aggressive histopathology, such as poorly differentiated endometrioid, and serous or clear cell carcinoma (Bokhman, 1983; Rose *et al.*, 2017). These patients account for the majority who present with stage III or IV disease that are typically nonresponsive to the gold standard chemotherapy treatment involving carboplatin plus paclitaxel, which portends a poor prognosis (Creutzberg *et al.*, 2004; Rose *et al.*, 2017; Young *et al.*, 2015). The identification of novel treatment modalities for patients diagnosed with biologically aggressive EC remains an unmet medical need.

Antibody–drug conjugates (ADCs) precisely target tumor cells as they are composed of a monoclonal antibody specific to surface antigens present on particular tumor cells and then kill these tumor cells with a highly potent chemical linker. ADCs may therefore optimize tumor targeting *in vivo* while potentially minimizing the side effects of highly toxic chemotherapy agents (Tsimberidou, 2015). Many ADCs are currently in late‐stage development, while others are either in clinical trials or have recently been approved for clinical use by the Food and Drugs Administration (FDA). For example, T‐DM1 (Kadcyla; Genentech/Roche, South San Francisco, CA, USA) is currently approved by the European Medical Agency (EMA) and FDA for patients with HER2‐positive metastatic breast cancer. Additionally, IMGN853 (Immunogen, Waltham, MA, USA) has already demonstrated high preclinical activity against type II endometrial cancer and other solid tumors (Ab *et al.*, 2015; Altwerger *et al.*, 2018; English *et al.*, 2014; Nicoletti *et al.*, 2015).

Human trophoblast cell‐surface antigen 2 (Trop‐2) is a 45 kDa transmembrane glycoprotein encoded by the *TACSTD2* gene on chromosome 1p32, which is differentially expressed in a variety of epithelial tumors (Cardillo *et al.*, 2015). Trop‐2 overexpression has been previously demonstrated to represent an independent marker for poor prognosis in multiple human tumors including endometrial endometrioid adenocarcinomas by promoting increased invasion and metastases (Bignotti *et al.*, 2012; Trerotola *et al.*, 2013). Importantly, the differential expression in tumor cells when compared to normal tissues renders Trop‐2 an attractive target for cancer immunotherapy.

Sacituzumab govitecan or IMMU‐132 (SG) is a novel ADC combining the humanized RS7 antibody targeting Trop‐2 coupled to a hydrolyzable linker that allows for a time‐dependent release of the payload, SN‐38, the active metabolite of irinotecan (7‐ethyl‐10‐hydroxycamptothecin) to the tumor tissue (Goldenberg *et al.*, 2015). SN‐38 has a 100‐ to 1000‐fold higher potency than irinotecan (Goldenberg *et al.*, 2015; Liu *et al.*, 2009; Mathijssen *et al.*, 2001). SG is characterized by a conjugation of drug to monoclonal antibody at a high ratio (8 : 1) without affecting antibody targeting and pharmacokinetics. Importantly, the pH‐sensitive hydrolyzable linker of SG allows for a strong, time‐released bystander effect against nearby Trop‐2‐negative tumor cells within the tumor microenvironment (Goldenberg *et al.*, 2015). Currently, no reports exist in the literature on the potential clinical activity of SG in EC patients. However, encouraging clinical activity of SG has recently been reported in a phase I/II IMMU‐132‐01 basket study of small‐cell lung cancer and non‐small‐cell lung cancer as well as in studies of triple‐negative breast cancer, HR+/HER2‐ breast cancer, and urothelial cancer (Bardia *et al.*, 2019; Faltas *et al.*, 2016; Gray *et al.*, 2017; Heist *et al.*, 2017).

The initial objective of this study was to determine the expression of Trop‐2 in EC tissue samples and primary established EC cell lines. Thereafter, we aimed to examine the preclinical antitumor activity of SG against multiple primary EC cell lines and xenograft models.

## 2 Materials and methods

### 2.1 Establishment of EC cell lines

The Institutional Review Board (IRB) approved this study, and patient consent was obtained per institutional guidelines prior to tissue collection. The EC cell lines were established from fresh tumor biopsy samples and de‐identified as described previously from our group (El‐Sahwi *et al.*, 2010; Roque *et al.*, 2014; Zhao *et al.*, 2013). As this study involves work performed with human samples, all the study methodologies conformed to the standards set by the Declaration of Helsinki (World Medical Association, 2001).

### 2.2 Tissue microarray

A retrospective cohort of stage I‐IV endometrioid EC tissue microarray (TMA) format samples were used in this study (*n* = 143). Representative areas from primary EC were selected in hematoxylin/eosin‐stained preparations by a trained gynecologic surgical pathologist, and 0.6 mm cores were obtained and arrayed in a recipient block. In order to increase representation and capture possible marker heterogeneity, two cores were obtained from different areas of each tumor were included in the TMA samples. Sections of the resultant TMA samples were cut at 5 μm and then transferred to glass slides for further histologic staining and processing. The tissue samples were collected with either specific consent or a waiver of consent under an approved Yale Human Investigation Committee protocol. Next, purified goat polyclonal antibody against the recombinant human Trop‐2 extracellular domain (R&D Systems, Inc., Minneapolis, MN, USA; diluted 1 : 100) was applied for 1 h. After that, a secondary biotinylated anti‐goat antibody (Vector Laboratories, Burlingame, CA, USA; diluted 1 : 250) and the streptavidin–biotin complex (StreptABComplex/HRP; Dako, Carpinteria, CA, USA) were applied. Subsequently, 303‐diaminobenzidine (Dako) was used as a chromogen and the sections were counterstained by hematoxylin (Dako). For all of the above, appropriate positive and negative controls were used. The percentage of tumor cells with membranous Trop‐2 immunoreactivity was estimated, and the staining intensity was measured semiquantitatively using the following scale: 0 for no staining; 1+ for weak; 2+ for moderate; and 3+ for strong staining. In order to obtain the final immunoreactivity score, staining intensity (1+, 2+, 3+) was multiplied by the percentage of positive tumor cells. The final immunoreactivity score was then classified into four ordinal categories: 0–9 negative (score 0), 10–99 weak (score 1), 100–199 moderate (score 2), and 200–300 strong (score 3).

### 2.3 Determination of Trop‐2 expression in primary EC cell lines

Flow cytometry to determine Trop‐2 expression was performed on primary poorly differentiated EC cell lines that were cultured *in vitro* for up to 50 passages. EC cell lines were incubated with 2.5 μg·mL^−1^ of unconjugated antibody hRS7 IgG for 120 min at 4 °C, and then stained with a fluorescein isothiocyanate‐conjugated goat anti‐human F(ab’)2 immunoglobulin (FITC) that was used as a secondary reagent (BioSource International, Camarillo, CA, USA). The data were acquired using cell quest software (BD Biosciences, San Diego, CA, USA). Mean fluorescence intensity (MFI) was evaluated using cell quest and prism 8. Cell lines with MFI greater than 100 were determined to have 3+ expression of Trop‐2 and with MFI of 51–100 2+, 21–50 1+, and 20 or less were considered negative for Trop‐2 expression.

### 2.4 Drugs

Sacituzumab govitecan (hRS7‐CL2A‐SN‐38), a nontargeting control ADC (h679‐CL2A‐SN‐38), and unconjugated monoclonal antibody hRS7 IgG were obtained from Immunomedics, Inc. (Morris Plains, NJ, USA). Lyophilized SG and control ADC were dissolved in sterile 0.9% sodium chloride as a 2 μm stock solution for the *in vitro* experiments. Drug‐to‐antibody ratio (DAR) of SG and control ADC was 6.78 and 6.84, respectively. For the *in vitro* experiments, the dosage of the drug was adjusted according to the DAR, in order to expose cells treated with SG and control ADC to equivalent quantities of SN‐38. For *in vivo* experiments, SG and the control ADC were reconstituted in sterile 0.9% sodium chloride as a 5 mg·mL^−1^ solution. hRS7 IgG (molecular weight: 150 kDa) was obtained in liquid form from Immunomedics, Inc., as a 10 mg·mL^−1^ solution.

### 2.5 Antibody‐dependent cellular cytotoxicity (ADCC)

Standard 4‐h chromium (^51^Cr) release assays were performed in order to measure the cytotoxic reactivity of Ficoll–Hypaque‐separated peripheral blood lymphocytes (PBLs), in combination with the drug SG, the control ADC, and the hRS7 IgG against the EC cell lines at an effector to target ratio (*E* : *T*) of 5 : 1 and 10 : 1. The release of ^51^Cr from target cells was measured as evidence of tumor cell lysis after exposure of the tumor cells to a concentration of 2.5 µg·mL^−1^ of SG, the control ADC, or hRS7 IgG. Negative controls for this experiment included tumor cells incubated with PBLs and no ADCs. The positive control condition included 1% SDS to achieve complete lysis of target cells. Chimeric anti‐CD20 mAb rituxan (2.5 μg·mL^−1^) was used in all bioassays as a negative control for hRS7 IgG. The release of ^51^Cr by cytolysis from the target cells was counted in a gamma radiation counter (2470 WIZARD2 Automatic Gamma Counter; PerkinElmer, Waltham, MA, USA). The percentage cytotoxicity of SG, the control ADC, or hRS7 IgG was calculated by the following formula: % cytotoxicity = 100 × (*E* − *S*)/(*T* − *S*), where *E* is the experimental release, *S* is the spontaneous release by target cells, and *T* is the maximum release by target cells lysed with 0.1% SDS. The results were reported as mean ± SEM.

### 2.6 Flow cytometry‐based cytotoxicity

Each of the EC cell lines tested was plated at a density of 30 000–80 000 cells/well in six‐well tissue culture plates with RPMI 1640 media supplemented with 10% FBS, 1% amphotericin, and 1% penicillin/streptomycin. Cells were incubated at 37 °C and 5% CO_2_ for 24 h after which they were treated with SG, the control ADC, and hRS7 IgG at the following concentrations of 0.2, 0.5, 1, 2, 4 nm. The concentration of the control ADC was adjusted based upon its DAR to assure the EC cells were treated with an equal amount of SN‐38. Cells were exposed to the drugs for 10 h before being washed with culture medium to remove any unbound ADC or unconjugated mAb. Then, the six‐well plates were incubated for an additional 72 h after which the cells were harvested, centrifuged, and stained with propidium iodide (2 µL of 500 µg·mL^−1^ stock solution in PBS). The viable cells were then quantified using a flow cytometry‐based assay that has been previously characterized (Roque *et al.*, 2014). A minimum of three independent experiments per cell line were performed in order to determine the IC50’s of SG, the control ADC, and hRS7 IgG in EC cell lines.

### 2.7 Bystander effect

ARK4, a Trop‐2‐negative uterine serous cell line stably transfected with a green fluorescence protein (GFP) plasmid (pCDH‐CMV‐MCSEF1‐copGFP, a gift from Simona Colla, MDACC) was plated (80 000 cells/well) alone in a 6‐well plate (2 mL per well). A 1 : 1 ratio of this ARK4 cell line stably transfected with GFP was admixed with END(K)265, a 3+ Trop‐2 expressing EC cell line, and then plated in a 6‐well plate (2 mL per well). After overnight incubation, cells were treated with either SG or the control ADC at a concentration of 1 nm for 10 h. Cells were then washed to remove unbound conjugate, and after additional 72 h, cells were collected, centrifuged, and stained with propidium iodide (2 µL of 500 µg·mL^−1^ stock solution in PBS) to identify the percentage of live versus dead cells that were present in each well. The bystander effect was assessed by comparing the percentage of live Trop‐2‐negative cells (GFP‐ARK4) when they were cocultured with Trop‐2‐positive cells (ARK2) and treated with either SG versus the control ADC.

### 2.8 *In vivo* testing

The *in vivo* antitumor activity of SG, the control ADC, and hRS7 IgG was tested in xenograft models using the Trop‐2 + END(K)265 cell line, a grade 3 EC with mixed endometrioid and clear cell histology. Each mouse (female, of age 5–8 weeks old with severe combined immunodeficiency (SCID); ENVIGO, Indianapolis, IN, USA) received a subcutaneous injection of 14 million END(K)265 cells suspended in 300 µL of a 1 : 1 solution of sterile PBS containing cells and Matrigel^®^ (BD Biosciences). When a tumor volume of 0.3 cm^3^ was obtained, the mice were then randomized into four groups (six mice/group): saline control, SG, control ADC, and hRS7 IgG. SG, the control ADC, and hRS7 IgG were given at a dose of 500 μg in 100 μL IV twice per week for three weeks by retro‐orbital and/or tail vein injection. Tumor volume was measured twice weekly using Vernier calipers. Determination of tumor volume was obtained using the formula (*A* ^2^ × *B*)/2, where *B* represented the largest tumor diameter size and *A* was the smallest perpendicular tumor diameter. The mice were subsequently euthanized when the group's average tumor volume reached 1.5 cm^3^. All animal care and euthanasia were carried out according to the regulations and rules established by the Institutional Animal Care and Use Committee (IACUC), which are in accordance with National Institutes of Health Guide for the Care and Use of Laboratory Animals.

### 2.9 Statistical analysis

Statistical analysis and IC_50_ extrapolations were performed using graphpad prism 8 (GraphPad Software, Inc. San Diego, CA, USA). Five drug concentration points were used for the calculation of IC_50_ in agreement with the previous literature (Turner and Charlton, 2005). The differences in the inhibition of proliferation in the EC cell lines as a result of exposure to the various treatments were evaluated using two‐tailed unpaired Student's *t*‐test. The differences in tumor volumes at specific time points were compared using an unpaired *t*‐test. Overall survival data were analyzed using the Kaplan–Meier method, and survival curves were compared using the log‐rank test. A two‐sided *P*‐value < 0.05 was considered to be statistically significant.

## 3 Results

### 3.1 Determination of Trop‐2 expression in EC patient samples by TMA

**Figure 1:**
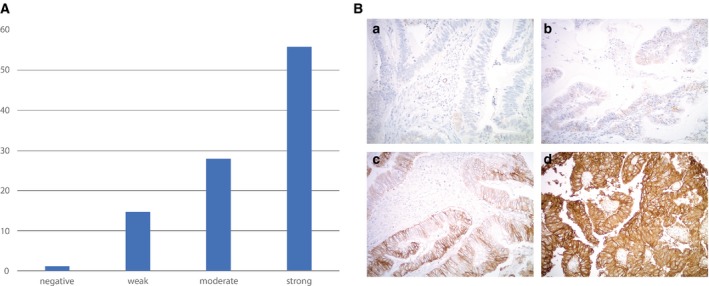
(A) Tissue microarray of 143 endometrioid EC samples. (B) Representative IHC images from the tissue microarray: (A) no Trop‐2 immunostaining (score 0), (B) weak focal (score 1), (C) moderate focal (score 2), and (D) strong diffuse (score 3) Trop‐2 expression. All images at 200× original magnification.

Semiquantitative analysis of Trop‐2 expression by immunohistochemistry (IHC) was performed using a TMA containing 143 EC patient samples. Moderate‐to‐strong (score 2–3) Trop‐2 expression was found in 84% (120/143) of these tumors (Fig. 1A and Table 1). In Fig. 1B, representative IHC images are presented.

**Table 1:** Semi‐quantitative analysis of Trop‐2 expression by IHC.

TROP‐2 expression	Count	%
Negative	2	1.3
Weak	21	14.7
Moderate	40	28
Strong	80	56
total	143	100.0

### 3.2 Determination of Trop‐2 expression in primary EC cell lines by flow cytometry

**Figure 2:**
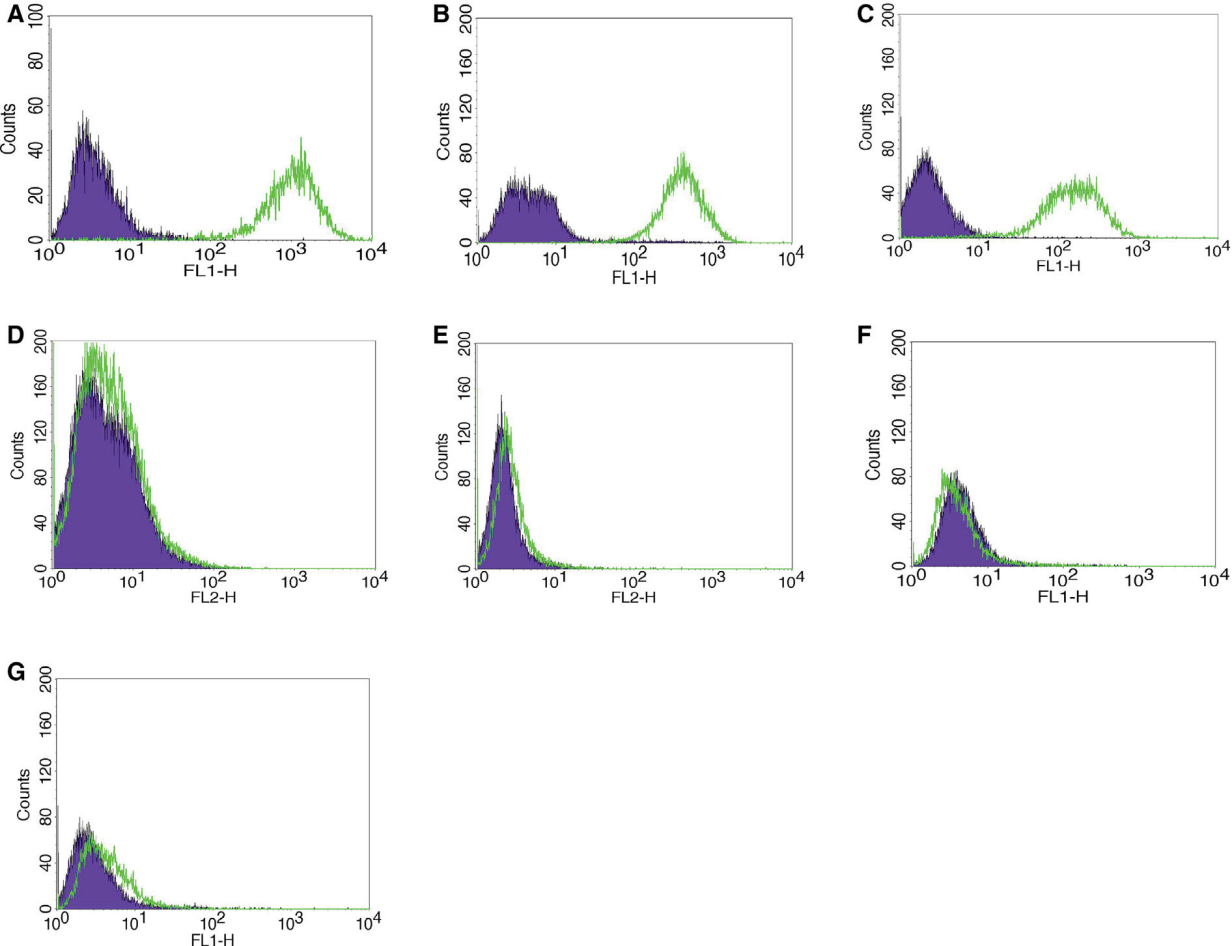
Representative flow cytometry histograms of the seven primary EC cell lines. (A) END(K)254, (B) END(K) 82, (C) END(K)265 cell lines demonstrating 3+ Trop‐2 expression and (D) END(K)283, (E) END(K)361, (F) END(K)181, and (G) END(K)23 cell lines demonstrating 0 Trop‐2 expression.

The expression of Trop‐2 was evaluated in seven primary EC cell lines. Table 2 demonstrates the clinicopathologic data of the patients from which these primary EC cell lines were established, which includes the cancer histology, stage, and grade clinic–pathologic and primary site of the tumors. With the use of flow cytometric analysis, three out of seven (43%) of the EC cell lines were found to have 3+ Trop‐2 expression (Table 2). Figure 2 demonstrates the flow cytometric analysis of the seven primary EC cell lines comparing those cell lines with 3+ Trop‐2 expression (i.e., END(K)254, END(K) 82, END(K)265 cell lines) versus those with 0 Trop‐2 expression (i.e., END(K)283, END(K)361, END(K)181, and END(K)23).

**Table 2:** Endometrial cancer cell lines with demographics, stage, histologic grade, primary site of tumor, MFI, and score for Trop‐2. MFI 0–20 = flow cytometry score 0; MFI 21–50 = flow cytometry score 1+; MFI 51–100 = flow cytometry score 2+; MFI > 100 = flow cytometry score 3+. International Federation of Gynecology and Obstetrics (FIGO) staging and grading.

Endometrioid endometrial cancer cell lines							Flow cytometry score
Cell line	Age	Ethnicity	FIGO stage	Primary site	Histology	∆TROP2 MFI	
END(K)23	66	Black	IIIA	Endometrium	Endometrioid G3	0.0	0
END(K)254	65	White	IIIC	Endometrium	Endometrioid G3	1208.9	3+
END(K)181	88	White	II	Endometrium	Endometrioid G3	0.0	0
END(K)82	58	White	IIIC	Endometrium	Endometrioid G2\G3	450.2	3+
END(K)283	70	White	IIIC	Endometrium	Endometrioid G3	2.06	0
END(K)265	50	White	IIIA	Endometrium	Endometrioid G3\CC	186.2	3+
END(K)361	65	white	X^a^	Endometrium	Endometrioid G3	1.6	0

a Tumor collected from surgical specimen after neoadjuvant chemotherapy treatment.

### 3.3 SG and hRS7 IgG mediate strong ADCC against Trop‐2‐positive primary EC

**Figure 3:**
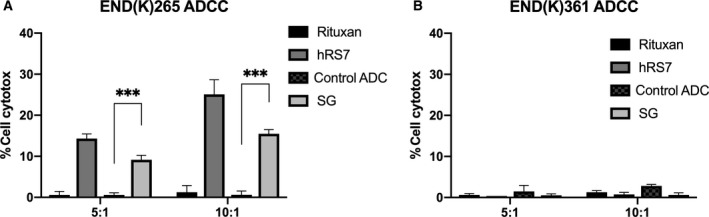
Antibody‐dependent cellular cytotoxicity results (mean ± SEM) of SG, control ADC, hRS7 IgG, and rituximab (anti‐CD20) in two representative EC cell lines [i.e., (A) END(K)265 3+ Trop‐2 cell line vs (B) END(K)361 Trop‐2‐ cell line] in the presence of PBL. Significant ADCC was detectable only against the Trop‐2 + tumors (*P* < 0.001).

A Trop‐2‐positive cell line (i.e., END(K)265) and Trop‐2‐negative cell line (i.e., END(K)361) were both tested for their potential sensitivity to PBL‐mediated cytotoxicity using standard 4‐h ^51^Cr release assays. It was found that when both EC cell lines were combined with isotype control antibody (rituxan) (2 μg·mL^−1^) at *E*:*T* ratios of 5 : 1 and 10 : 1, they were resistant to PBL‐mediated cytotoxicity (Fig. 3). However, treatment with equivalent concentrations (2 μg·mL^−1^) of hRS7 IgG, SG, and the control ADC revealed that hRS7 IgG and SG were highly effective in inducing ADCC against primary EC cell lines expressing Trop‐2 at high levels (i.e., in the END(K)265 cell line) but the control ADC did not. As shown in Fig. 3A, at *E*:*T* ratios of 5 : 1 and 10 : 1, a mean cytotoxicity ± SEM = 12.3 ± 3.1% for SG and 19.7 ± 5.4% for hRS7‐IgG was determined, respectively, while low/negligible killing (i.e., 0.5 ± 0.03%) was induced by control ADC (*P* < 0.001 at 5 : 1 and 10 : 1 ratio, Fig. 3A). In contrast, there was no significant ADCC against the Trop‐2‐negative cell line END(K)361 after treatment with hRS7 IgG, SG, and the control ADC (Fig. 3B).

### 3.4 *In vitro* viability assays with SG, the control ADC, and hRS7 IgG in primary EC cell lines

**Figure 4:**
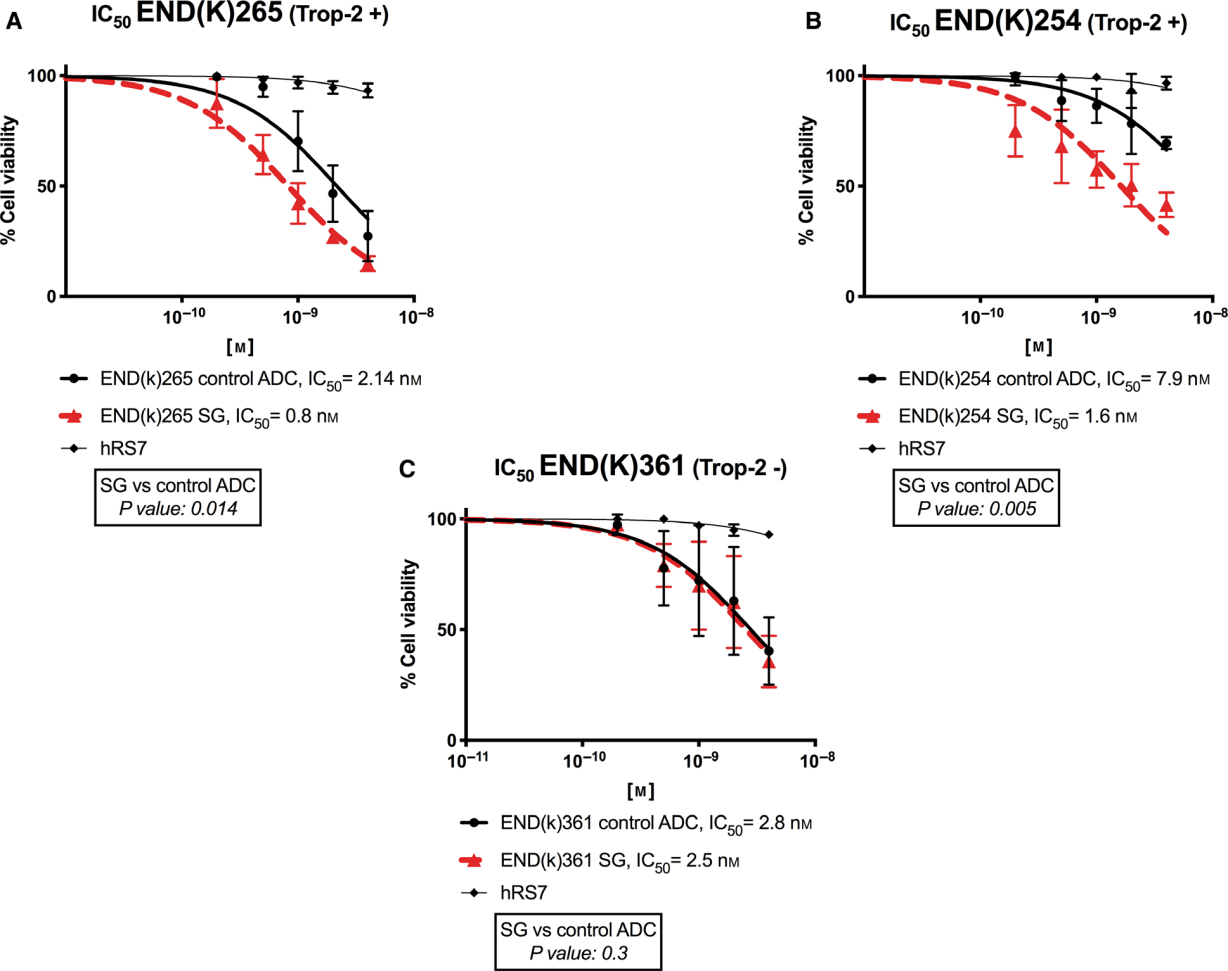
Determination of SG, control ADC, and hRS7 IgG IC_50_ (mean ± SEM) in primary EC cell lines. (A, B) EC cell lines with high Trop‐2 expression (3+) (i.e., END(K)265, END(K)254) demonstrated significantly lower IC_50_ when compared to control ADC (*P* < 0.05). (C) EC cell line with low/negligible Trop‐2 expression (i.e., END(K)361) showed no difference in the IC_50_s of SG and control ADC. hRS7 IgG antibody was inactive against all cell line tested. A minimum of three experiments for each cell line were performed.

Subsequently, three primary EC cell lines (two were Trop‐2‐positive and one was Trop‐2‐negative) were treated with scalar concentrations of SG, the control ADC, and hRS7 IgG. There was a significant increase in cell cytotoxicity induced against Trop‐2‐positive EC cell lines treated with SG (i.e., a 2.7‐fold, 4.9‐fold increase against END(K)265 and END(K)254, respectively) than that of those treated by the control ADC (*P* = 0.014 and *P* = 0.005, respectively) (Fig. 4A,B). In contrast, no differences were found in Trop‐2‐negative END(K)361 EC cell lines (*P* = 0.3) (Fig. 4C). In the absence of PBL, the IC50 of hRS7 IgG was extrapolated using the statistical software of graphpad prism 8 to be greater than 40‐fold higher than IC50 of SG for all cell lines (data not shown).

### 3.5 Bystander effect *in vitro*

**Figure 5:**
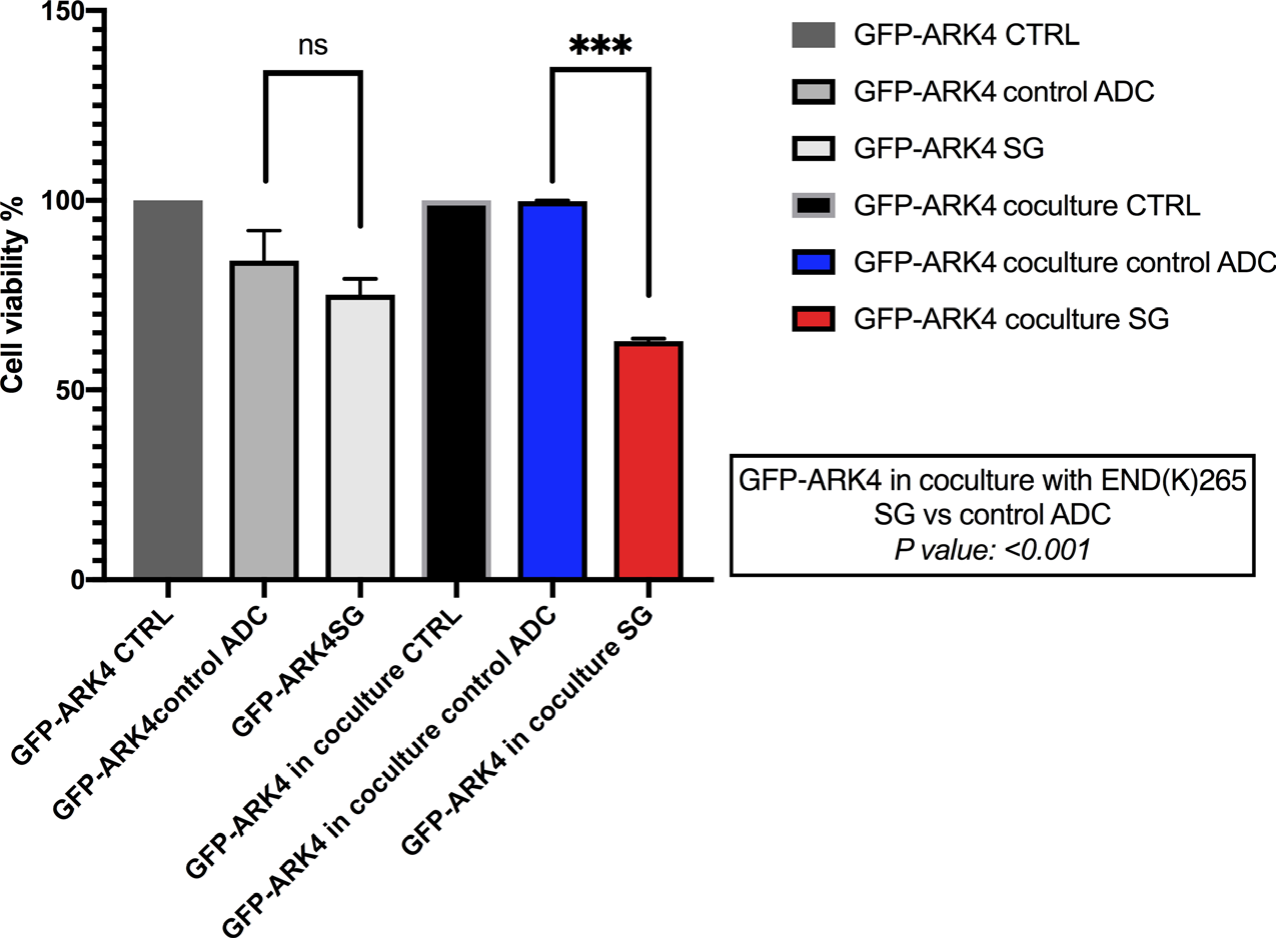
Bystander assay. Black bar: low/negative Trop‐2 expressing ARK4 cells (GFP‐ARK4 cells), cocultured with END(K)265 (i.e., 3+ Trop‐2) without treatment. Blue bar: low Trop‐2 expressing cells (GFP‐ARK4) cocultured with high Trop‐2 expressing cells (END(K)265) and treated with control ADC at 1 nm concentration. Red bar: low Trop‐2 expressing cells (GFP‐ARK4) cocultured with high Trop‐2 expressing cells (END(K)265) and treated with SG at 1 nm concentration. Significant increase in GFP‐ARK4 cell cytotoxicity was detected at the time of the co‐incubation with END(K)265 cells, when they were treated with SG (*P* < 0.05). Error bars indicate standard deviation (SD) throughout figure.

In order to evaluate the potential of SG to mediate a bystander killing effect toward EC cell lines with heterogeneous Trop‐2 expression, we admixed cells with high Trop‐2 expression (i.e., END(K)265 cells) and those with low or negligible Trop‐2 expression (i.e., GFP‐ARK4 cells) for 72 h. As shown in Fig. 5, there was a significant increase in the cytotoxicity of ARK4 cells when ARK4 and END(K)265 were cultured together and treated with SG when compared to ADC control‐treated cocultures (Fig. 5, *P* = 0.001).

### 3.6 Antitumor activity of SG in the xenograft model

**Figure 6:**
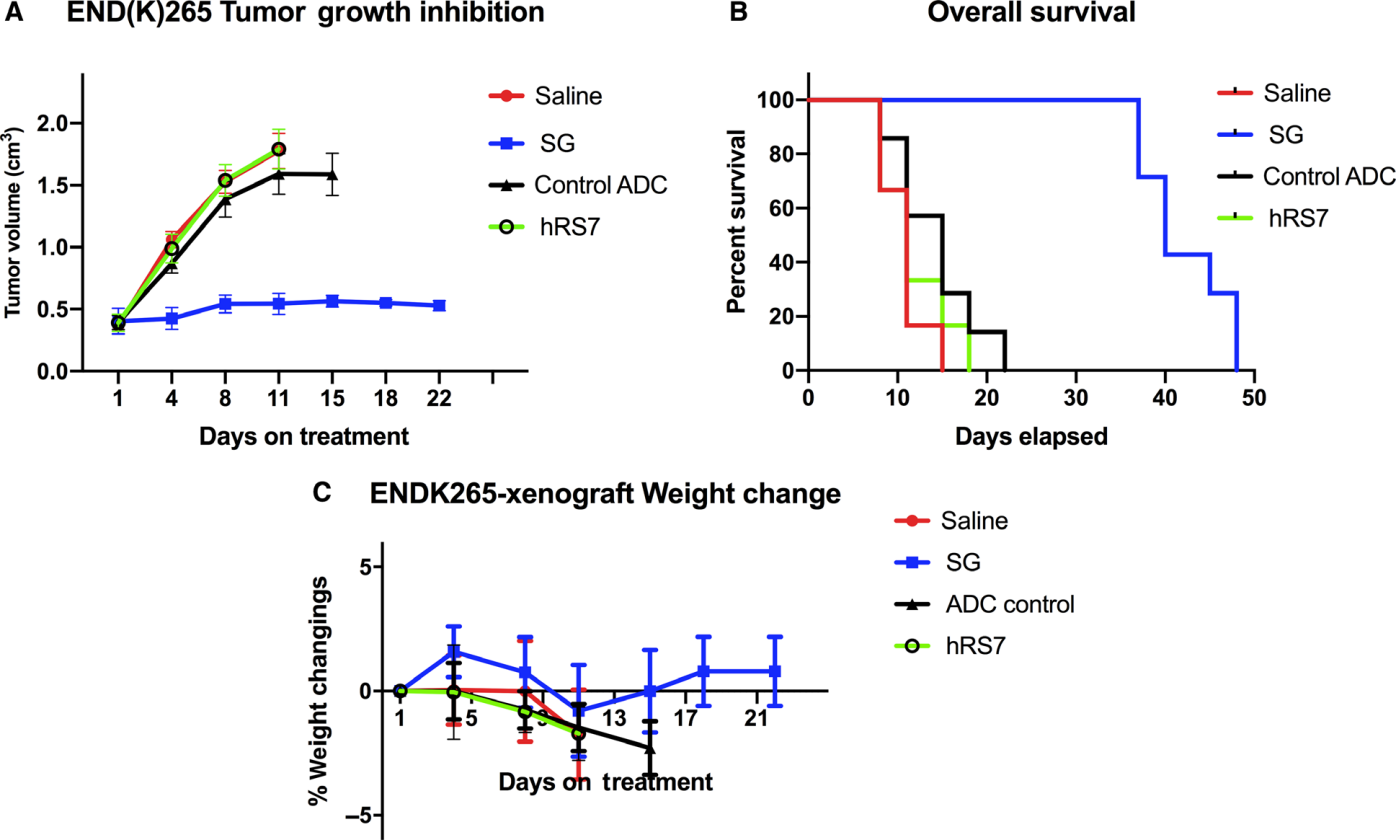
*In vivo* efficacy of SG: (A) antitumor activity and (B) overall survival of SG were compared to controls including control ADC, unconjugated mAb (hRS7 IgG), and saline, in EC xenograft models (i.e., END(K)265, 3+ Trop‐2‐positive). (C) Mice weight change during treatment. Mice were treated intravenously with twice‐weekly doses for 3 weeks as described in Methods. (A) A significant difference in tumor growth inhibition was detected beginning on day 4 (*P* < 0.05) in the SG‐treated group when compared to the other control groups. (B) Median survival for the SG group was 40 days, compared to 15 days for control ADC and 11 days for hRS7 and saline. Error bars indicate standard deviation (SD) in both (A) and (C).

Due to the high Trop‐2 expression as well as the consistent engraftment of primary END(K)265 tumor cells in SCID mice, these xenograft models were chosen for the *in vivo* experiments comparing the potential antitumor activity of SG, the control ADC, hRS7 IgG, and saline. Within 4 days of therapy initiation (i.e., after only one injection), SG demonstrated a significant antitumor effect in these chemotherapy‐resistant tumors compared to all controls, including nonspecific ADC (Fig. 6A; *P* = 0.011). This SG‐mediated inhibition of tumor growth was evident at all subsequent time points up to day 15 when the control ADC animals were euthanized secondary to progression of disease, which was defined as a tumor volume greater than 1.5 cm^3^ (*P* < 0.01). This antitumor effect translated into a significant survival benefit for SG‐treated animals in comparison with all the controls (Fig. 6B; *P* < 0.05). Median survival for the SG group was 40 days versus 15 days for the control ADC, and 11 days for hRS7 IgG and saline (Fig. 6B). Overall, the mice tolerated the treatment well and there was no significant difference in the weight of the mice among all four groups (Fig. 6C).

## 4 Discussion

High expression of Trop‐2 has been discovered in a variety of human epithelial tumors including cervical, uterine, and ovarian cancers and confers tumor cells with an increased ability to proliferate and migrate (Bignotti *et al.*, 2012; Liu *et al.*, 2013; Santin *et al.*, 2004; Varughese *et al.*, 2011b; Xu *et al.*, 2017). Importantly, as a result of its differential overexpression in tumor cells when compared to normal cells, Trop‐2 may represent an excellent target for targeted therapeutics such as ADCs.

Accordingly, in this study, we evaluated the level of Trop‐2 expression in 143 ECs and evaluated the potential cytotoxicity that treatment with SG may induce against multiple primary EC cell lines with different degrees of Trop‐2 expression. Moderate‐to‐strong Trop‐2 expression was found in 84% (120/143) of the EC samples evaluated by IHC and 43% of the primary EC cell lines assessed by flow cytometry. Importantly, we have shown Trop‐2‐positive EC cell lines to be highly sensitive to treatment with SG when compared to treatment with the control ADC and hRS7 IgG. In contrast, no differences were found in Trop‐2‐negative cell lines. These results demonstrate the need for Trop‐2 receptor expression on tumor cells for the induction of SG cytotoxic activity against EC.

Furthermore, we demonstrated that the mechanism by which SG induces tumor cell killing is not only due to the internalization of the ADC and the consequent intracellular release of the toxic payload SN‐38 but it may also be potentially mediated by immune system cells (i.e., NK cells). Indeed, both SG and hRS7 IgG demonstrated significant induction of ADCC toward Trop‐2‐positive EC cell lines in the presence of PBL, while the control ADC generated only a low level of cytotoxicity. The killing activity was Trop‐2‐specific as demonstrated by the negligible ADCC induced by SG against Trop‐2‐negative tumors. These findings support previous results from our group evaluating the activity of the unconjugated humanized anti‐Trop‐2 monoclonal antibody hRS7 in ovarian cancer (Bignotti *et al.*, 2012; Trerotola *et al.*, 2013; Varughese *et al.*, 2011a).

EC is a heterogeneous disease, and accordingly, expression of Trop‐2, similarly to other surface markers, may not be uniformly expressed. Importantly, due to the cleavable hydrolyzable linker of SG the toxic payload, SN‐38 may be released in tumors both intracellularly and extracellularly in the tumor microenvironment. Therefore, this ultimately permits the delivery of a therapeutic concentration of the drug to surrounding cells, which were not the initial target of the ADC (Goldenberg *et al.*, 2015). Consistent with this view, SG may kill Trop‐2‐positive tumor cells by intracellular intake of SN‐38, while neighboring cells (i.e., Trop‐2‐negative tumor and/or endothelial/stromal cells) may be killed by the extracellular release of SN‐38 (Goldenberg *et al.*, 2015). To validate this hypothesis, we performed *in vitro* experiments admixing Trop‐2‐positive with Trop‐2‐negative tumor cells before exposing them to SG. We consistently found SG to induce killing against Trop‐2‐negative tumor cells but only when they were admixed with Trop‐2 overexpressing tumor cells. Overall, these results strongly support the fact that SG may also be effective in the treatment of patients with recurrent EC whose tumors often possess varying degrees of Trop‐2 expression.

Nonetheless, the *in vivo* experiments with Trop‐2‐positive EC xenografts established from a patient with a biologically aggressive tumor (i.e., mixed clear cell and G3 endometrioid histology) demonstrated that a few injections of SG were highly effective in inducing regression of EC xenografts. Indeed, twice‐weekly administration of SG demonstrated a statistically significant difference in tumor growth inhibition of END(K)265 when compared to administration of the control ADC. It was considered that a possible limitation in the use of SG in the clinical setting is its potential toxicity. Thus, it is important to note that there was no evidence of acute or chronic toxicity detected in the animals treated with SG for the entire duration of the study. These results are the first to demonstrate the efficacy and safety profile of the *in vivo* activity of SG against biologically aggressive and poorly differentiated EC. These findings warrant the support of the use of SG in clinic trials.

Consistent with our preclinical *in vitro* and *in vivo* results in biologically aggressive endometrial cancer, results from a phase I/II clinical study reported acceptable toxicity and encouraging therapeutic activity of SG against multiple recurrent human epithelial cancers including urothelial cancers and lung cancers (Bardia *et al.*, 2019; Faltas *et al.*, 2016; Gray *et al.*, 2017; Heist *et al.*, 2017; Starodub *et al.*, 2015). Moreover, our group has recently reported an impressive clinical response to SG in a 74‐year‐old woman with recurrent/chemotherapy‐resistant endometrial serous tumor overexpressing Trop‐2 (66% reduction of target lesions by RECIST 1.1 criteria for over 10 months of follow‐up) (Han *et al.*, 2018). Furthermore, other encouraging results of treatment with SG were previously described in a cohort of 108 heavily pretreated metastatic triple‐negative breast cancers. These patients had an objective response rate of 33.3%, median response duration of 7.7 months, and a clinical benefit rate of 45.4% (Bardia *et al.*, 2019). As a result of these encouraging clinical findings in breast cancer patients, SG has received breakthrough therapy designation from the FDA for the treatment of TNBC patients with metastatic disease who have failed at least two prior therapies.

## 5 Conclusions

Trop‐2 overexpression is found in over 80% of EC, and our *in vitro* studies demonstrate that SG proves to be highly cytotoxic to these primary EC cell lines. Furthermore, SG induces significant ADCC against Trop‐2‐positive EC cells in the presence of effector cells (NK cells) but more uniquely SG demonstrates a significant bystander killing effect, which may aid in the treatment of tumors with heterogeneous antigen expression. Nonetheless, EC xenografts overexpressing Trop‐2 were found to be highly sensitive to treatment with SG. Our results combined with recent phase II data demonstrating significant clinical responses in tumors resistant to standard chemotherapy regimens support the implementation of clinical trials for patients with recurrent and poorly differentiated EC that harbor Trop‐2‐positive tumor expression.

## Conflict of interest

All authors fulfill the conditions required for authorship. ADS declares research funding support to Institution from ImmunoMedics. AM declares that she receives research funding support from Spanish Medical Oncology Society.

## Author contributions

ADS designed research; EP, SL, BZ, SB, AB, LZ, AM, EB, PM, NB, GA, CH, GM, ER, D‐AS, MA, JTR performed research; PES, GS contributed new reagents/analytic tools, ADS and EP wrote the paper.
